# Zinc-Dependent Transcriptional Regulation in *Paracoccus denitrificans*

**DOI:** 10.3389/fmicb.2017.00569

**Published:** 2017-04-11

**Authors:** Durga P. Neupane, Belkis Jacquez, Anitha Sundararajan, Thiruvarangan Ramaraj, Faye D. Schilkey, Erik T. Yukl

**Affiliations:** ^1^Department of Chemistry and Biochemistry, New Mexico State UniversityLas Cruces, NM, USA; ^2^National Center for Genome ResourcesSanta Fe, NM, USA

**Keywords:** zinc, gene regulation, RNA-seq, transcription factors, metal homeostasis

## Abstract

Zinc homeostasis is critical for bacterial survival and is mediated largely at the transcriptional level by the regulation of zinc uptake and efflux genes. Here we use RNA-seq to assess transcriptional changes as a result of zinc limitation in the denitrifying bacterium *Paracoccus denitrificans*. The results identify the differential expression of 147 genes, most of which were upregulated in zinc-depleted medium. Included in this set of genes are a large number of transition metal transporters, several transcription factors, and hypothetical proteins. Intriguingly, genes encoding nitric oxide reductase (*norCB*) and nitrite reductase (*nirS*) were also upregulated. A Zur consensus binding motif was identified in the promoters of the most highly upregulated genes. The zinc uptake regulator (Zur) from this organism was also characterized and shown to bind to the Zur motif in a zinc-dependent manner. This work expands our current understanding of the transcriptional response of gram-negative bacteria to zinc limitation and identifies genes involved in denitrification as part of the Zur regulon.

## Introduction

Zinc, an essential trace element, is indispensable for the survival of all organisms and mediates crucial roles as a catalytic center of many enzymes and in organizing protein structure (Coleman, 1992). However, if present in excess, zinc is toxic to the cell due to its high affinity for divalent metal binding sites and displacement of elements such as manganese or iron required for protein function (Waldron and Robinson, 2009). Hence, physiological homeostasis of intracellular zinc within a narrow range is requisite for survival and is ensured by highly regulated zinc uptake (Hantke, 2005) and efflux systems (Nies, 2003). In pathogenic bacteria, zinc homeostasis is particularly important. Invading bacteria may encounter severe zinc limitation as a result of “nutritional immunity” (Weinberg, 1975; Hood and Skaar, 2012), where zinc and manganese are sequestered by the host protein calprotectin in response to infection (Clohessy and Golden, 1995; Damo et al., 2013; Kehl-Fie et al., 2013). On the other hand, the host may also implement zinc toxicity as a microbial defense mechanism (Neyrolles et al., 2013; Ong et al., 2014). Therefore, an understanding of the mechanisms of bacterial zinc homeostasis may provide a means of bacterial pathogen control.

Bacterial zinc homeostasis is largely controlled at the transcriptional level through the actions of zinc-responsive regulators of zinc uptake and efflux genes (Choi and Bird, 2014; Capdevila et al., 2016). In many bacteria, the zinc uptake regulator (Zur) is the predominant regulator of zinc uptake genes, typically ATP binding cassette (ABC) transporter operons, repressing their expression when bound to zinc (Fillat, 2014). In *Xanthomonas campestris*, zinc-bound Zur can also act to activate expression of a zinc efflux pump (Huang et al., 2008). In *Escherichia coli*, a separate regulator ZntR activates the expression of *zntA*, which encodes a zinc efflux pump of the P-type ATPase family (Brocklehurst et al., 1999). In either case, the combined action of zinc-responsive activators and repressors establishes a “set point” (Outten and O'Halloran, 2001) of intracellular zinc concentrations determined by the metal binding affinity of the regulators.

The Zur and ZntR regulons have largely been established through transcriptional studies comparing WT and deletion strains. Relatively few studies have investigated global transcriptional changes in response to zinc limitation. Among these, non-specific chelators such as N,N,N′,N′-tetrakis(2-pyridylmethyl)ethane-1,2-diamine (TPEN) are often used due to the difficulty in removing zinc from culture media. TPEN binds other divalent elements such as cadmium, cobalt, nickel, and copper with higher affinity than zinc (Anderegg et al., 1977), resulting in changes in gene expression that are not specifically related to zinc deprivation (Sigdel et al., 2006; Graham et al., 2009). Therefore, many of the gene expression changes observed under these conditions should not be attributed as resulting solely from zinc limitation. To our knowledge, global transcription changes in response to zinc limitation alone have been investigated for only a few WT bacterial species including *E. coli* (Graham et al., 2009), *Neisseria meningitides* (Pawlik et al., 2012), *Pseudomonas protegens* Pf-5 (Lim et al., 2013) and *Streptococcus pneumoniae* (Shafeeq et al., 2011). These investigations demonstrate differential regulation of no more than ~100 genes, which invariably include ATP binding cassette (ABC) transporters for zinc. These are under direct transcription control of Zur, which also typically regulates its own transcription. The Zur regulon often includes various other genes, including those for non-zinc parologs of ribosomal proteins, other transcription factors and hypothetical proteins (Panina et al., 2003; Owen et al., 2007; Shin et al., 2007; Gabriel and Helmann, 2009; Li et al., 2009; Schröder et al., 2010; Pawlik et al., 2012; Lim et al., 2013; Mortensen et al., 2014; Pederick et al., 2015). Recent ChiP-on-chip experiments identified Zur binding sites within or near a number of genes involved in oxidative or disulfide stress response in *Bacillus subtilis* (Prestel et al., 2015). These studies highlight the diversity of functions regulated by Zur in response to zinc limitation among different bacterial species.

In this study, we have employed RNA-seq to explore changes in global gene expression as a result of zinc limitation in *Paracoccus denitrificans*. *P. denitrificans* is a gram-negative bacterium known for denitrification (reduction of nitrate) and general metabolic versatility. We have previously identified two distinct zinc-specific ABC transporter operons, *znuABC* and *aztABCD* (Handali et al., 2015a,b) in this organism. The latter has only recently been described and is highly conserved in human pathogens including *Klebsiella pneumonia* and *Nocardia farcinica*. Thus, the mechanisms of zinc homeostasis in *P. denitrificans* may be relevant to bacterial pathogenesis. Further, we identified a link between zinc and nitrogen metabolism where zinc depletion leads to upregulation of genes involved in denitrification. Comparison of promoter regions of upregulated genes allowed for the identification of a conserved Zur consensus sequence that was subsequently confirmed by electrophoretic mobility shift assay (EMSA) using purified *P. denitrificans* Zur.

## Materials and methods

### Bacterial strain and growth conditions

*P. denitrificans* strain PD1222 was grown in minimal media with defined composition adapted from Graham et al. (2009) and Wang et al. (2003). Bulk media containing 40 mM MES, 20 mM KCl, 60 mM NH_4_Cl, 0.5 g/L yeast extract was dissolved in MilliQ water, adjusted to pH 7.4 and passed through a column of Chelex resin followed by supplementation with 134 μM EDTA, 250 μM CaCl_2_, 810 μM MgSO_4_ before autoclaving. Solutions of 1.0 M succinate and 764 mM beta-phosphoglycerate were prepared separately, passed through a column of Chelex resin and autoclaved. All media solutions were stored in acid washed bottles to minimize contaminating metal ions. Stock metal salt solutions of 100 mM ZnSO4, 47 mM MnSO_4_, 53 mM NaMoO_4_, 1.6 mM CuCl_2_, 20 mM Fe-citrate (5.5 g/L FeSO4.7H2O, and 5.35 g/L citric acid) were prepared separately and filter sterilized. Complete minimal media was made by combining above components to final concentrations of 38 mM MES, 19 mM KCl, 57 mM NH_4_Cl, 0.47 g/L yeast extract, 126 μM EDTA, 236 μM CaCl_2_, 764 μM MgSO_4_, 50 mM succinate, 7.6 mM beta-phosphoglycerate, 47 μM MnSO_4_, 53 μM NaMoO_4_, 1.6 μM CuCl_2_, 20 μM Fe-citrate and 0–50 μM ZnSO_4_.

Cells grown overnight in media containing 10 μM Zn at 30°C were pelleted, washed with minimal media lacking zinc and then used to inoculate three different growth conditions differing in added ZnSO_4_. Zn-replete media contained 50 μM ZnSO_4_, Zn-depleted media contained no added ZnSO_4_ and Zn-chelated media was made by the addition of 50 μM TPEN to Zn-depleted media. Cell growth in all conditions was monitored spectrophotometrically at 600 nm using an Agilent Cary 60 UV-Vis spectrophotometer. For RNA extraction, 5 ml cells at mid exponential growth phase (OD_600_ ~0.4–0.5) were harvested from each condition and 2 ml of chilled 5% w/v phenol in ethanol was added immediately to preserve RNA. The cells were incubated on ice for 30 min and 1.4 ml aliquots centrifuged and stored at −80°C prior to RNA isolation. RNA was extracted from three replicates from each growth condition using a PureLink® RNA Mini Kit (Ambion®). DNA contamination was removed using an on-column DNAse digestion protocol (Invitrogen®). RNA concentration and purity were determined spectrophotometrically using a Nano drop Spectrophotometer ND-1000.

### cDNA synthesis and qRT-PCR

cDNA was synthesized from 500 ng of pure RNA in 20 μl reaction volume using iScript™cDNA synthesis kit (Bio-Rad®). cDNA was diluted with nuclease-free water to a final concentration 10 ng/μl and used for real time PCR reactions. The primers (Table S1) were designed to amplify 100–150 base pairs (bp) of target genes with an average Tm ~55°C and were used at a final concentration 0.3 μM. Quantification of amplified PCR product using Power SYBR® Green PCR Master Mix (Applied Biosystems) was monitored by CFX96 real-time system combined with a C1000 Thermal cycler (Bio-Rad). The relative expression of genes was normalized to *dnaN* (pden0970), a housekeeping gene encoding the β-subunit of DNA-polymerase III previously used for real-time PCR experiments in this organism (Sullivan et al., 2013).

### RNA-Seq

Nine (three growth conditions, three replicates as stated above) RNA libraries were prepared using the standard Illumina TruSeq library kit. Prior to library preparation, ribosomal RNA was removed using the ribozero rRNA removal kit (bacteria) by Illumina. Libraries were then prepared and sequenced on Illumina HiSeq 2000 instrument to generate 50 bp single end reads. Sequence reads were subjected to post processing to trim Illumina adapters and primer sequences.

### Transcriptome analysis

High quality reads for each sample were aligned to the *P. denitrificans* PD1222 genome, downloaded from GenBank repository (GCA_000203895.1). Associated annotation file in GFF format was used to obtain genic information for downstream analysis. Alignments were generated using GSNAP (version released on 2014_12_29) with the following parameters; indel penalty = 2, maximum mismatches = 0.06 and everything else set to default (Wu and Nacu, 2010). Read counts were generated using NCGR's in house pipeline, Alpheus (Miller et al., 2008). Gene expression for each sample was computed as a measure of the total number of reads uniquely aligning to the reference, binned by genic coordinates (information acquired from the annotation file). Differential gene expression analysis was performed using the Bioconductor package DESeq (Anders and Huber, 2010). Raw read counts thus obtained were normalized to account for differences in sequencing depth and composition using methods implemented within DESeq. Differential expression of pairwise comparisons (of the different conditions) was assessed using the negative binomial test with the Benjamani–Hochberg false discovery rate (FDR) adjustment (Hochberg and Benjamini, 1990) applied for multiple testing corrections. For this study, an FDR of 0.05 was applied and any candidate that had a *p*-adjusted value of ≤ 0.05 was considered to be significantly up- or down-regulated.

### Cloning, heterologous expression, and purification of *Zur*

The entire *zur* gene (pden4139) was PCR amplified by primer sets, FWD: 5′-ACTATCATATGCCCACTTCGGAATCCCCG-3′ and REV: 5′-ACTATGGATCCTCACAGGCCGGCCTC-3′ and cloned into a pMAL-c5X vector (New England BioLabs) at BamHI and NdeI restriction sites. Plasmid was transformed into a BL21 derived cell line (NEB® Express) and grown in LB media with 100 μg/ml ampicillin at 37°C, 250 rpm to an OD_600_ ~ 0.6. Protein expression was induced by addition of IPTG to 1.0 mM and further incubated at 18°C, 225 rpm for 12–14 h. Cells were harvested by centrifuging at 4,000 rpm for 20 min at 4°C. Cell pellets obtained from 1 L bacterial culture were re-suspended in 50 ml lysis/equilibration buffer composed of 20 mM Tris, pH 7.4, 200 mM NaCl and 1 mM EDTA. The cells were lysed by sonication and cell debris was removed by centrifugation at 20,000x g for 20 min at 4°C. The cleared lysate was applied to an amylose resin column, washed four times with lysis buffer and eluted with lysis buffer containing 10 mM maltose. The purified fusion protein was dialyzed at 4°C overnight against 1 L 20 mM Tris pH 8.0, 100 mM NaCl to remove excess maltose and EDTA.

The MBP fusion tag was cleaved by addition of Factor Xa protease (NEB) to fusion protein at a ratio of 1:100 by mass in the presence of 2 mM CaCl_2_. The reaction mixture was incubated for 90 min at room temperature and the MBP fusion tag and factor Xa removed by anion exchange chromatography on a HiTrap Q HP column (GE Healthcare). The column was equilibrated with 20 mM Tris, pH 8.0 and proteins eluted on a linear gradient from 150 to 350 mM NaCl. Zur eluted at ~230 mM NaCl and was highly pure as judged by SDS-PAGE. The Zur-containing fractions were collected and concentrated, and the protein concentration measured by Bradford method (Bradford, 1976). The Zur oligomerization state was determined by applying the cleavage reaction mixture to a Hi-Prep Sephacryl S-100 HR column (GE Healthcare) equilibrated with 20 mM Tris pH 8.0, 150 mM NaCl and calibrated using proteins ranging in size from 6.5 to 75 kDa from a gel filtration calibration kit (GE Healthcare).

### Zinc quantitation and generation of Apo-Zur

Apo-Zur was generated by dialysis of the purified protein against 50 mM NaOAc buffer pH 4.5, 50 mM EDTA, and 150 mM NaCl followed by a final dialysis against 20 mM tris buffer pH 8.0, 150 mM NaCl, and 3.4 g/L Chelex resin (Biorad). The protein concentration was again measured by Bradford assay. Protein samples 10–20 μM were digested in 4 M HNO_3_ overnight at 70°C and diluted 2.5-fold with MilliQ water prior to metal analysis. For media and buffer samples, 2.0 mL were combined with 0.5 mL concentrated HNO_3_ and digested overnight at 70°C. Metal content was quantified using a Perkin-Elmer 2100 DV inductively coupled plasma—optical emission spectrometer (ICP-OES), calibrated with a multielement standard (Alpha Aesar) at a wavelength of 213.857 nm. All samples were run in triplicate.

### Equilibrium dialysis

Purified Zur at 10 μM was combined with 20 μM ZnCl_2_ in a final volume of 2 mL and incubated for 30 min at room temperature. The sample was then dialyzed against 1 L 20 mM tris pH 8.0, 150 mM NaCl containing 1 μM ZnCl_2_ at 4°C overnight. Aliquots of protein and dialysis buffer were taken for ICP-OES analysis as described above. Remaining protein was transferred to fresh buffer with no added zinc, dialyzed and analyzed as previously. After compensating for dilution, the free zinc concentration was determined by ICP-OES of dialysis buffer and subtracted from the total zinc concentration in the protein samples to calculate protein-bound zinc.

### Metal binding affinity

The affinity of apo Zur for zinc was evaluated using a competitive fluorescence assay with magfura-2 (MF-2; Thermofischer scientific) as described by Golynskiy et al. (2006). The fluorescence excitation from 250 to 450 nm was measured at an emission wavelength of 510 nm using a Varian Cary Eclipse fluorescence spectrophotometer, with entrance and exit slits set at 10 nm. MF-2 concentration was measured spectrophotometrically using an extinction coefficient at 369 nm of 22,000 M^−1^cm^−1^ (Golynskiy et al., 2006). Prior to each experiment, apo Zur was desalted into HEPES binding buffer (20 mM HEPES pH 7.2, 200 mM NaCl and 5% glycerol (v/v) buffer that had been passed through a Chelex column to remove trace metals) and its concentration determined as above. Final concentrations of 1.0 μM apo Zur and 0.5 μM MF-2 were combined in 200 μL HEPES binding buffer. This mixture was titrated with 0.2 μM additions of ZnCl_2_ with the total titrant volume never exceeding 5% (v/v). The experiment was done in triplicate and fluorescence intensity change at 330 nm was fit using Dynafit (Kuzmic, 1996, 2009) to determine the dissociation constant (K_d_) value of Zur for zinc.

### Circular dichroism (CD)

CD spectra were recorded at 25°C using a Jasco-810 spectropolarimeter with a cuvette chamber regulated by a PTC-4235 Peltier device (Jasco). Apo Zur was diluted to 15 μM in 5 mM HK_2_PO_4_ pH 8.0, 150 mM NaCl in a 1 mm quartz cuvette. 37.5 μM ZnCl_2_ was added for measurements on the holo protein. Spectra were acquired from 190 to 260 nm at 1 nm bandwidth, 2 s response time, 0.5 nm data pitch and 10 nm/min scan speed. Each spectrum is the average of three accumulations and a buffer blank was subtracted. Y-axis values have been converted to mean residue ellipticity. For thermal stability experiments, the intensity at 208 nm was monitored from 25 to 90°C every 0.2°C with a constant heating rate of 1.0°C/min. The fraction of folded protein at a given temperature T was determined using the following equation (Dow et al., 2015):

$$\begin{array}{l} \text{Fraction Folded}=\frac{\theta_{T}-\;\theta_{25}}{\theta_{25}-\;\theta_{90}} \end{array}$$

Values of θ at 25 and 90°C were chosen to correspond to 100 and 0% folded states, respectively. Spectra were fit to estimate secondary structure contributions on the online server Dichroweb (Whitmore and Wallace, 2004) using the CDSSTR algorithm (Sreerama and Woody, 2000).

### Electrophoretic mobility shift assay (EMSA)

One hundred twenty to one hundred fifty bp of promoter DNA from genes of interest were amplified by specific primers listed in Table S1. The amplified product was assessed by 1% agarose gel electrophoresis and was purified by Pure Link™ Quick PCR product purification kit (Invitrogen). EMSA reactions, adapted from Sheehan et al. (2015), were carried out in 50 μl reaction volume containing 10 mM Tris, pH 8.0, 10 mM NaCl, 100 mM potassium glutamate, 1.0 mM DTT, 50 mM KCl, 1.07 μg/ml BSA, 10 μg/ml (0.1 μM) promoter DNA, 0–20 μM Zur, 25 μM ZnCl_2_, and 100 μM EDTA. The final three elements were added in the order indicated to allow zinc to bind Zur before addition of EDTA. Fifty microgram per milliliter of salmon sperm DNA was included in some reactions to test binding specificity. Competition assays were also performed using 5 μM of specific or random 59 bp oligomer competitors. The specific competitor contained two Zur binding motifs as in the *zur/znuA* promoter or the degenerated sequence in the *norC* promoter. The random competitor was a scrambled sequence (Figure S1). The reaction mixtures were incubated for 30 min at room temperature and ficoll was added to 3% prior to loading the sample onto 8% native PAGE gel. The gel was pre-run without sample for 1 h at 145 V, 100 mA current in 90 mM Tris-boric acid buffer, pH ~8.3. Fifty microliters of sample was loaded and run for 4 h at 4°C as described above. Gels were stained with ethidium bromide for 1 h prior to UV imaging. For the metal specific Zur-DNA EMSA experiment, 10 μM apo-Zur protein was combined with 25 μM Zn^2+^, Mn^2+^, Fe^2+^, Ni^2+^, or Cu^2+^, followed by addition of 100 μM EDTA in EMSA buffer.

## Results and discussion

### RNA-Seq

**Figure 1:**
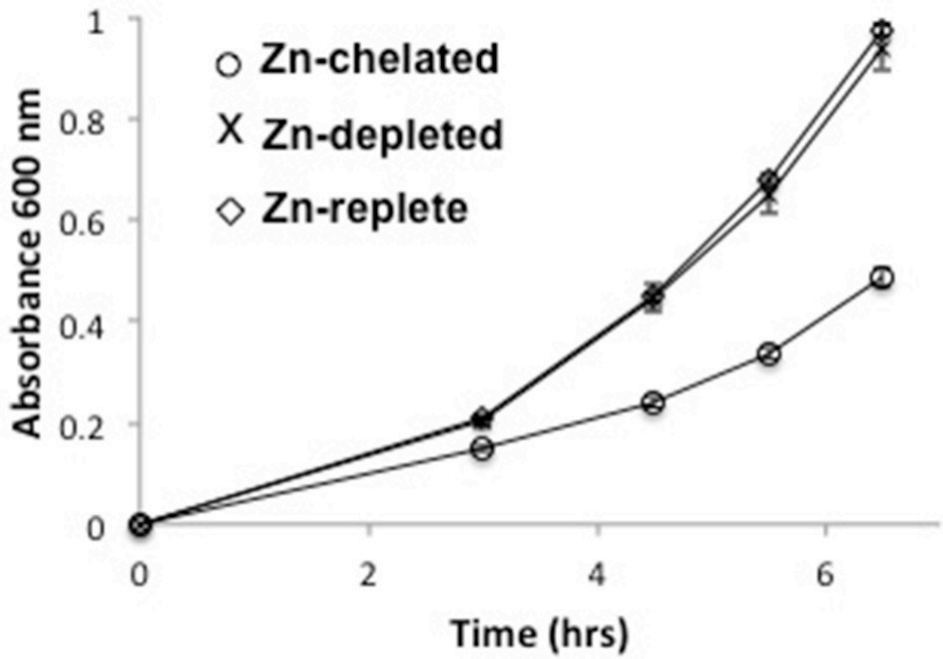
**Growth curves measured at 600 nm for *P. denitrificans* grown in Zn-replete, Zn-depleted or Zn-chelated media**. Error bars represent the standard deviation of measurements from three independent cultures.

Global gene expression changes in response to zinc availability were determined using RNA-seq of *P. denitrificans* cultures grown in defined media differing in the amount of available zinc. Generally, zinc concentrations above 100 μM activate expression of zinc efflux genes while those below 10 μM lead to derepression of zinc import genes (Hantke, 2005). Therefore, a concentration of 50 μM added zinc was chosen for our Zn-replete conditions to establish a baseline gene expression level. Zn-depleted media were made by simple omission of zinc from defined media. Despite efforts to exclude sources of exogenous zinc, ICP-OES of several Zn-depleted media preparations indicated an average zinc concentration of 1.1 μM. Based on the above argument, this concentration should still be sufficient to elicit a transcriptional response. Nevertheless, to induce severe metal starvation, we also grew cells in Zn-chelated media made by adding 50 μM TPEN to Zn-depleted media. Cell growth was only significantly inhibited in Zn-chelated conditions whereas cells in Zn-depleted media exhibited essentially identical growth rates to those in Zn-replete media (Figure 1), consistent with previous observation (Handali et al., 2015a).

Cells were harvested from mid-exponential growth phase (OD_600_ = 0.4–0.5) and RNA was purified for RNA-seq. Nine samples over all conditions (three replicates each) were sequenced on the Illumina HiSeq2000 platform to yield ~13.5–16.5 million uniquely aligning reads per sample. This coverage is sufficient to probe both highly as well as moderately expressed transcripts. High correlation existed between all three replicates within a sample, which was represented as a dendrogram, generated using a hierarchical clustering approach integrated as part of the DESeq tool.

Using zinc-replete conditions as a reference, we observe differential expression of 147 genes in Zn-depleted media and 1,341 genes in Zn-chelated media using a criterion of *p* < 0.05 to evaluate differential expression. The large number of differentially expressed genes in zinc-chelated media is likely due to non-specific chelation by TPEN, as discussed above. On the other hand, the relatively small number of genes differentially expressed under zinc-depleted conditions can be attributed to zinc limitation. Therefore, we have focused our analysis on the data obtained from Zn-depleted conditions, but a full list of differentially regulated genes in both conditions is available in Supplementary Information.

Of the 147 genes differentially expressed in zinc-depleted media, 133 were upregulated whereas only 14 were repressed. Many are organized into operons and have been categorized on the basis of their putative functions (Tables 1–**5**). By far the most abundant class of genes identified come from metal transporter systems (Table 1), particularly the ABC transporters. Among bacteria, high-affinity import of transition metals is primarily undertaken by ABC transporters, which are composed of a membrane spanning permease, a cytoplasmic ATPase and a periplasmic or lipoprotein solute binding protein (SBP). The SBPs confer high affinity and specificity to the transporter system and were originally grouped into eight clusters based on sequence homology, which also correlated closely with the type of substrate transported (Tam and Saier, 1993). A ninth cluster (cluster 9) was later proposed that was specific for transport of either zinc or manganese (Dintilhac and Claverys, 1997), with the presence of a region rich in His and Asp residues indicative of zinc specificity (Claverys, 2001). The SBPs were later classified according to structural similarity into six clusters (A-F), three of which were subdivided based on substrate type (Berntsson et al., 2010). Under this classification system, cluster A-I includes SBPs that directly bind iron, zinc or manganese.

**Table 1:** **Transporter genes: those in predicted operons are grouped within horizontal dividing lines**.

**Locus**	**Gene**	**Predicted Function**	**Fold change (Zn-depleted)**	**Fold change (Zn-chelated)**
pden0212	*zntA*	Heavy metal-transporting *P*-type ATPase	0.65	nd
pden1338^*^		Cobalamin synthesis protein P47K	13.2	9.8
pden1339		Hypothetical protein	10.6	9.6
pden1340		Hypothetical protein	8.6	8.3
pden1341^*^		Ni/dipeptide ABC transporter SBP	16	8.2
pden1342		Ni/dipeptide ABC transporter permease	13	7.7
pden1343		Ni/dipeptide ABC transporter permease	12.6	7.9
pden1344		Ni/dipeptide ABC transporter ATPase	14.8	9.0
pden1595^*^	*aztA*	Zn/Mn ABC transporter ATPase	14.8	9.4
pden1596	*aztB*	Zn/Mn ABC transporter permease	6.3	5.9
pden1597	*aztC*	Zn/Mn ABC transporter SBP	8.3	7.5
pden1598	*aztD*	Zn/Mn ABC transporter Zinc chaperone	24.5	27.6
pden4137	*znuB*	Zn/Mn ABC transporter permease	2.1	1.8
pden4138	*znuC*	Zn/Mn ABC transporter ATPase	2.3	2.0
pden4139^*^	*zur*	Zur transcriptional regulator	2.5	1.9
pden4140^*^	*znuA*	Zn/Mn ABC transporter SBP	11.5	6.4
pden4141		Hemin uptake protein HemP	4.2	1.5
pden4202		Hemin-degrading protein	3.8	3.3
pden4203		Fe(III)/B_12_ ABC transporter SBP	2.5	3.0
pden4204		Fe(III)/B_12_ ABC transporter permease	2.1	2.9
pden0296		TonB-dependent receptor	8.0	19.7
pden0299		Fe(III) ABC transporter permease	2.0	2.3
pden0300		Fe(III) ABC transporter SBP	3.0	2.2
pden0319		Mn/Fe transporter of NRAMP family	13.8	nd
pden0725		Biopolymer transporter TonB	2.1	nd
pden0726		Biopolymer transporter protein ExbD	2.4	nd
pden1367		B_12_ ABC transporter SBP	1.9	nd
pden1368		TonB-dependent receptor	2.2	1.6
pden1733		FTR1 iron permease	2.7	1.8
pden1842		Heavy metal transporting P-type ATPase	2.1	nd
pden2980		Fe(III)/B_12_ ABC transporter SBP	1.9	2.2
pden2981		Fe(III)/B_12_ ABC transporter permease	1.8	1.7
pden2982		Fe(III)/B_12_ ABC transporter ATPase	1.7	1.6
pden3010		Fe(III)/B_12_ ABC transporter SBP	1.9	2.3
pden3012		Fe(III)/B_12_ ABC transporter permease	1.7	1.8
pden3013		Fe(III)/B_12_ ABC transporter ATPase	2.5	2.0
pden3017		ABC transporter MDR/TAP subfamily	1.9	2.0
pden4169		Nitrate/sulfonate ABC transporter SBP	6.9	nd
pden4446		Fe(III) ABC transporter permease	1.8	2.1
pden4447		Fe(III) ABC transporter SBP	2.6	2.8
pden4448		Fe(III) ABC transporter ATPase	2.2	1.5
pden4830		Fe(III)/B_12_ ABC transporter permease	1.5	nd
pden5109		Arabinose ABC transporter permease	1.5	nd
pden5130		Iron dicitrate sensor FecR	2.3	2.0

nd, no differential expression detected.

* *Indicates the presence of a Zur motif (see Table 7) in the promoter region*.

Unsurprisingly, two zinc-specific cluster A-I (or cluster 9) ABC transporter operons *znuABC* and *aztABCD* were among the most strongly upregulated genes, consistent with previous qRT-PCR studies (Handali et al., 2015a,b). In fact, *aztD*, which encodes a periplasmic metallochaperone, exhibited nearly 25-fold induction under zinc-depleted conditions, the greatest change observed for any *P. denitrificans* gene. A seven-gene operon (pden1338–1344) was also strongly upregulated, with each member gene exhibiting >8-fold increase in expression. This operon contains a cluster C (or cluster 5) ABC transporter (pden1341–1344) with predicted specificity for nickel, di- and oligo-peptides, cellubiose, or Arg (Berntsson et al., 2010). It also contains a COG0523 gene annotated as a cobalamin synthase (pden1338). The COG0523 family genes have since been associated with zinc homeostasis (Haas et al., 2009; Capdevila et al., 2016) and are part of the Zur regulon for many species (Hood et al., 2012; Lim et al., 2013; Pederick et al., 2015; Nairn et al., 2016) The COG0523 proteins are metal-regulated GTPases (Blaby-Haas et al., 2012; Sydor et al., 2013), although their precise function is still unclear. Pden1338 is encoded in an operon together with two hypothetical proteins (pden1339 and 1340). A member of the NRAMP family of divalent metal transporters (pden0319) was also strongly upregulated as was a predicted TonB dependent receptor (pden0296) and a predicted SBP for nitrate/sulfonate (pden4169). A large number of predicted cluster A-1 and A-II ABC transporters of Fe, cobalamin or potentially heme were modestly upregulated between 1.5- and 4-fold, as were several other TonB receptor homologs. Finally, a predicted heavy metal transporting P-type ATPase was among the few genes downregulated in response to zinc limitation. This gene is 44% identical to the *E. coli* ZntA zinc efflux pump described above. Since the genome of *P. denitrificans* encodes several MerR family transcription factors homologous to ZntR, it is likely that one of these activates *zntA* expression under high zinc conditions as has been described in *E. coli* (Brocklehurst et al., 1999).

Interestingly, we did not see any greater induction of transporter genes in Zn-chelated medium. In fact, most exhibit slightly smaller fold changes in this condition, suggesting that zinc concentrations in the low micromolar range are sufficient to fully derepress these genes. Exceptions include the TonB dependent receptor (pden0296), which is upregulated nearly 20-fold in Zn-chelated media compared to 8-fold in Zn-depleted media and the NRAMP homolog (pden0319), which is not differentially expressed in Zn-chelated media.

An unexpected set of genes identified as upregulated by RNA-seq are involved in energy metabolism (Table 2), particularly denitrification and microaerobic oxygen respiration pathways. The *norCB* genes encode two subunits of nitric oxide reductase and *nirS* encodes the cytochrome *cd*_*1*_-type nitrite reductase (Stouthamer et al., 1997). The *norC* and *nirS* genes exhibit >10-fold increases in transcription in response to zinc limitation. Coupled with upregulation of a putative nitrate transporter (pden4169), this suggests a link between zinc availability and denitrification. We also observe modest upregulation of the *ccoNOQP* and *ccoGH*, encoding structural subunits of the cytochrome *cbb*_*3*_ oxidase and assembly factors, respectively (Pitcher and Watmough, 2004). Expression of this oxidase has only previously been observed in response to microaerobic conditions.

**Table 2:** **Energy metabolism: those in predicted operons are grouped within horizontal dividing lines**.

**Locus**	**Gene**	**Predicted function**	**Fold change (Zn-depleted)**	**Fold change (Zn-chelated)**
pden0655		Nitrite/sulphite reductase	1.8	nd
pden1843	*ccoH*	FixH family protein	2.4	nd
pden1844	*ccoG*	FixG Fe-S protein	3.7	nd
pden1845	*ccoP*	Cytochrome c oxidase, cbb3-type subunit III	2.3	nd
pden1846	*ccoQ*	Cytochrome c oxidase, cbb3-type subunit IV	2.4	nd
pden1847	*ccoO*	Cytochrome c oxidase, cbb3-type subunit II	2.5	nd
pden1848	*ccoN*	Cytochrome c oxidase, cbb3-type subunit I	2.7	nd
pden2483	*norB*	Nitric oxide reductase subunit B	3.7	3.6
pden2484^*^	*norC*	Nitric oxide reductase subunit C	13.5	3.6
pden2487	*nirS*	Dissimilatory nitrite reductase, cd1-type	11.3	nd
pden2880		F0F1-ATPase subunit	0.60	nd
pden4443		ScoI/SenC copper chaperone (CCO maturation)	0.46	4.1
pden4444		Cu(I) binding protein (CCO maturation)	0.33	4.6

nd, no differential expression detected.

* *Indicates the presence of a possible Zur motif (see Table 7) in the promoter region*.

The remaining differentially expressed genes can be classified as transcriptional regulators, other metabolic genes and hypothetical proteins (Tables 3–5). Three transcriptional regulators in addition to *zur*, which is part of the *znuABC* operon, were found to be modestly upregulated. The metabolic genes identified are part of separate pathways and are only slightly upregulated. A number of hypothetical proteins showed similar levels of differential expression with the notable exception of pden4221, which is upregulated nearly 10-fold. Also known as *nosC*, it is part of the *nos* regulon that encodes the nitrous oxide reductase *nosZ*, which reduces N_2_O to N_2_ in the final step of denitrification (Zumft, 1997). Although no specific function has been assigned to this gene, deletion of *nosC* results in deregulation of *nosZ* transcription in response to Cu availability (Sullivan et al., 2013). This illustrates yet another link between dentirification and zinc limitation in *P. denitrificans*.

**Table 3:** **Transcription factors**.

**Locus**	**Gene**	**Predicted function**	**Fold change (Zn-depleted)**	**Fold change (Zn-chelated)**
pden0071		CopG family transcription regulator	0.54	nd
pden0318		GntR family transcription regulator	3.3	nd
pden0668		TetR family transcription regulator	2.6	nd
pden3520		AraC family transcription regulator	5.4	2.3

*nd, no differential expression detected*.

**Table 4:** **Other metabolic pathways**.

**Locus**	**Gene**	**Predicted function**	**Fold change (0 Zinc vs 50 Zinc)**	**Fold change (TPEN vs 50 Zinc)**
pden0542		Cell wall hydrolase	0.62	nd
pden0669		3-oxoacyl-ACP synthase	2.7	1.6
pden1689		Dihydropteridine reductase	4.1	nd
pden1851		Coproporphyrinogen III oxidase	4.4	nd
pden2382		Isochorismate synthase	2.4	nd
pden2984		SAM dependent methyltransferase type 11	1.6	1.5
pden3290		N-acetylglucosamine transferase	0.60	nd
pden4789		FMN reductase	0.37	nd

*nd, no differential expression detected*.

**Table 5:** **Hypothetical proteins**.

**Locus**	**Gene**	**Predicted function**	**Fold change (Zn-depleted)**	**Fold change (Zn-chelated)**
pden0066		Hypothetical protein	0.47	nd
pden0317		Hypothetical protein	0.64	nd
pden0320		Hypothetical protein	1.8	nd
pden0654		Hypothetical protein	1.9	nd
pden1736		Hypothetical protein	2.3	4.2
pden3635		Hypothetical protein	3.9	nd
pden4221	*nosC*	Hypothetical protein, NosC-like	9.4	nd
pden4788		Hypothetical protein	0.49	nd

*nd, no differential expression detected*.

In many species, paralogs of ribosomal proteins that do not require zinc are regulated by Zur and induced during zinc limitation, providing a mechanism to spare zinc for essential, zinc-dependent functions (Panina et al., 2003; Capdevila et al., 2016). We do not observe this for *P. denitrificans* in Zn-depleted media. Similarly, zinc-dependent regulation of ribosomal genes by Zur was neither predicted nor observed for various species of *Corynebacterium* (Schröder et al., 2010), nor was it observed in *Acinetobacter baumannii* (Mortensen et al., 2014). Thus, it would seem that zinc-dependent regulation of ribosomal proteins is not a universal feature among bacteria and that *P. denitrificans*, like several other species, lacks this regulatory function.

With the exception of *norB* and *norC* genes, very few of the non-transporter related genes differentially regulated in Zn-depleted conditions were identified in Zn-chelated conditions. The reasons for this are unclear. Energy metabolism genes are highly regulated and sensitive to a number of growth conditions and transcription factors. It is therefore somewhat puzzling that TPEN, which induces transcriptional changes to 1,341 genes, does not influence their expression. Perhaps compensatory changes are made by other regulatory mechanisms, or TPEN induces a more general stress response that does not include these genes.

### qRT-PCR

**Figure 2:**
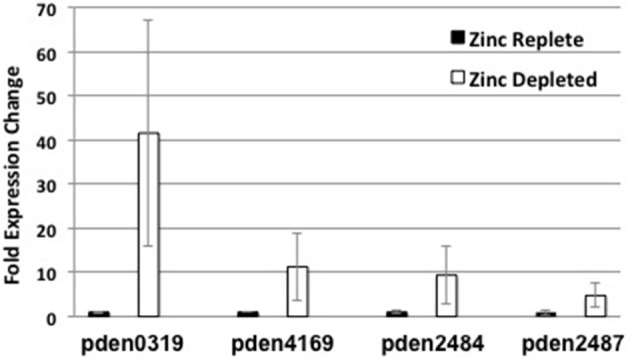
**qRT-PCR evaluation of relative expression levels of genes identified as upregulated by RNA-seq from samples grown in Zn-replete (*black bars*) or Zn-depleted (*white bars*) media**. *Error bars* represent the mean ± S.E. (*n* = 3). *Pden0319, pden4169, pden2484*, and *pden2487* encode NRAMP transporter, nitrate transporter, nitric oxide reductase, and nitrite reductase genes, respectively.

The upregulation of *znuA, aztC*, and *aztD* in response to zinc deficiency has been previously observed by qRT-PCR (Handali et al., 2015a,b). We used this technique to compare expression in Zn-depleted vs. Zn-replete conditions to confirm a subset of the most highly upregulated genes identified by RNA-seq (Figure 2). We observed fold increases in expression for the NRAMP family transporter (pden0319), nitrate/sulfonate ABC transporter SBP (pden4169), nitric oxide reductase subunit NorC (pden2484) and dissimilatory nitrite reductase NirS (pden2487) of 42, 11, 9, and 5, respectively. These are comparable to values obtained by RNA-seq in every case except for pden0319, which shows significantly greater induction by qRT-PCR. Importantly, upregulation of the unexpected nitrogen metabolism genes is independently confirmed by this technique.

### Characterization of Zur

*P. denitrificans* Zur protein was heterologously expressed as a fusion with maltose binding protein (MBP). The fusion protein was purified by amylose affinity chromatography and the MBP tag removed by protease Xa cleavage followed by ion exchange chromatography. Size exclusion chromatography demonstrated that Zur migrates exclusively as a dimer. As isolated, Zur contained 1.0 equivalents of zinc per monomer as determined by ICP-OES, indicating a single high-affinity zinc binding site. Apo-Zur was generated by dialysis as described in Section Materials and Methods, and ICP-OES confirmed that ~90% of zinc was removed by this procedure. A simple equilibrium dialysis experiment was performed to gain some insight into the affinity and stoichiometry of Zur for zinc. Briefly, a small volume of purified Zur was combined with a small excess of ZnCl_2_ and dialyzed against a large volume of buffer. After extensive dialysis, some of the protein and dialysis buffer were removed for ICP-OES analysis and the process repeated a second time with fresh buffer. The results indicate two zinc binding sites per Zur monomer (Table 6). One of these exhibits sub-micromolar affinity while the other has an apparent K_d_ in the low micromolar range.

**Table 6:** **Concentrations of zinc from Zur equilibrium dialysis experiments**.

**Dialysis**	**Protein-bound [Zn] (μM)**	**Free [Zn] (μM)**	**Zn:protein**
First	16.94	1.66	1.94
Second	11.01	0.92	1.24

**Figure 3:**
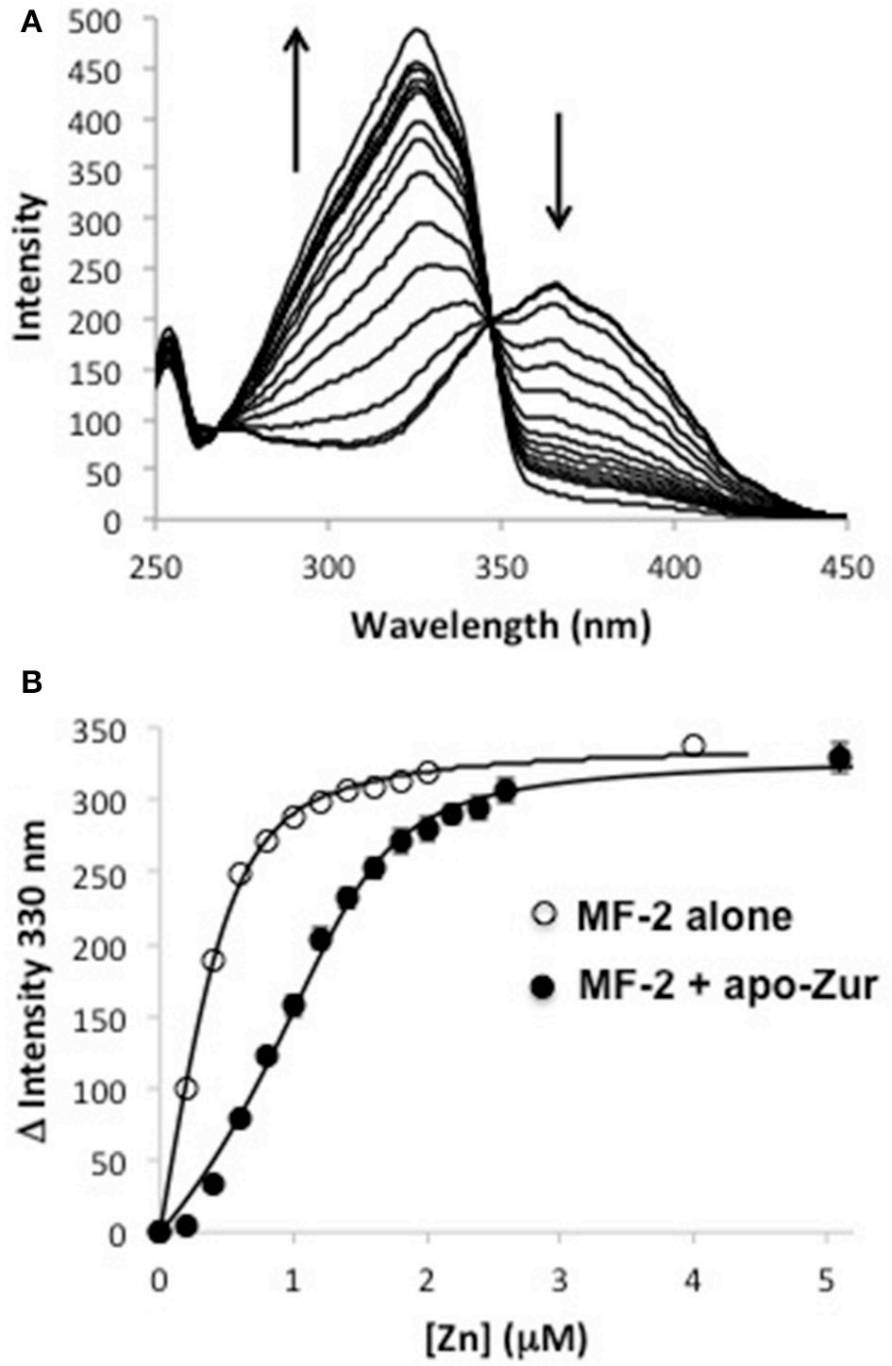
**Competitive binding assays for zinc with apo-Zur and MF-2. (A)** Representative fluorescence excitation spectra (λ_em_ = 510 nm) of 0.5 μM MF-2 and 1.0 apo-Zur titrated with increasing ZnCl_2_. Arrows indicate the direction of intensity change with increasing zinc. **(B)** Comparison of the intensity change at 330 nm for MF-2 alone or MF-2 + apo-Zur titrated with ZnCl_2_. Data for MF-2 alone is from a single experiment. Data for competitive binding is the average of three independent experiments, and error bars represent the mean ± S.E. (*n* = 3). Solid lines are least square fits of the data.

In order to refine these values, a competitive binding assay using the fluorescent probe magfura-2 (MF-2) was used (Golynskiy et al., 2006; Handali et al., 2015a,b). MF-2 alone exhibits a fluorescence excitation peak at ~360 nm and zinc binding shifts this peak to 330 nm (Figure 3A). Using the change in fluorescence at 330 nm, we determined a K_d_ for MF-2 in our buffer system of 100 nM, which is similar to the literature value 33 nM (Golynskiy et al., 2006; Figure 3B). Inclusion of apo Zur into the reaction shows that Zur competes effectively with MF-2 for zinc binding. Using the determined K_d_ value for MF-2, the competition data (an average of three independent experiments) fit well to a single binding site model for Zur with K_d_ = 40 ± 4 nM. The second Zur binding site is likely of too weak an affinity to effectively compete with MF-2 and is not included in fits to the data. Combined with equilibrium dialysis, we ascribe a lower limit for the K_d_ of this site at 1 μM.

**Figure 4:**
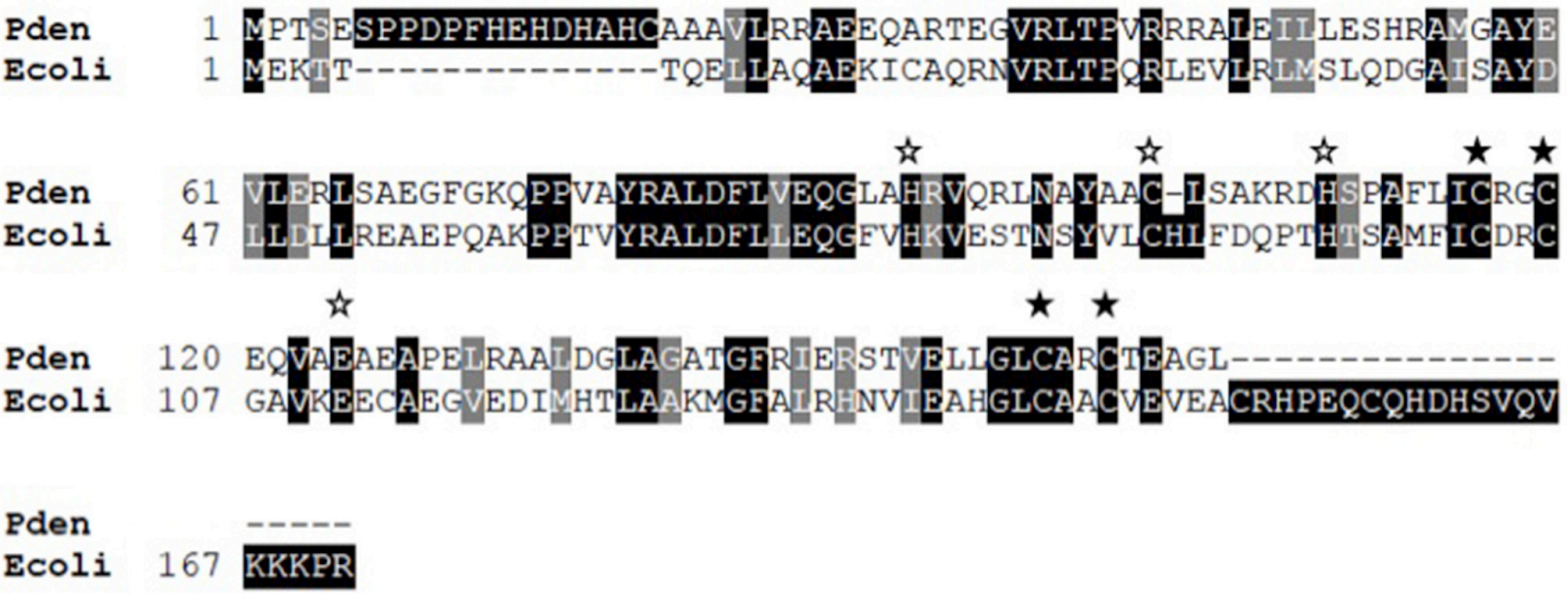
**Sequence alignment of Zur homologs from *P. denitrificans* (Pden) and *E. coli* (Ecoli)**. Identical residues are shaded in black, similar residues in gray. Filled stars mark residues comprising the zinc binding A site while open stars mark those of the B site.

**Figure 5:**
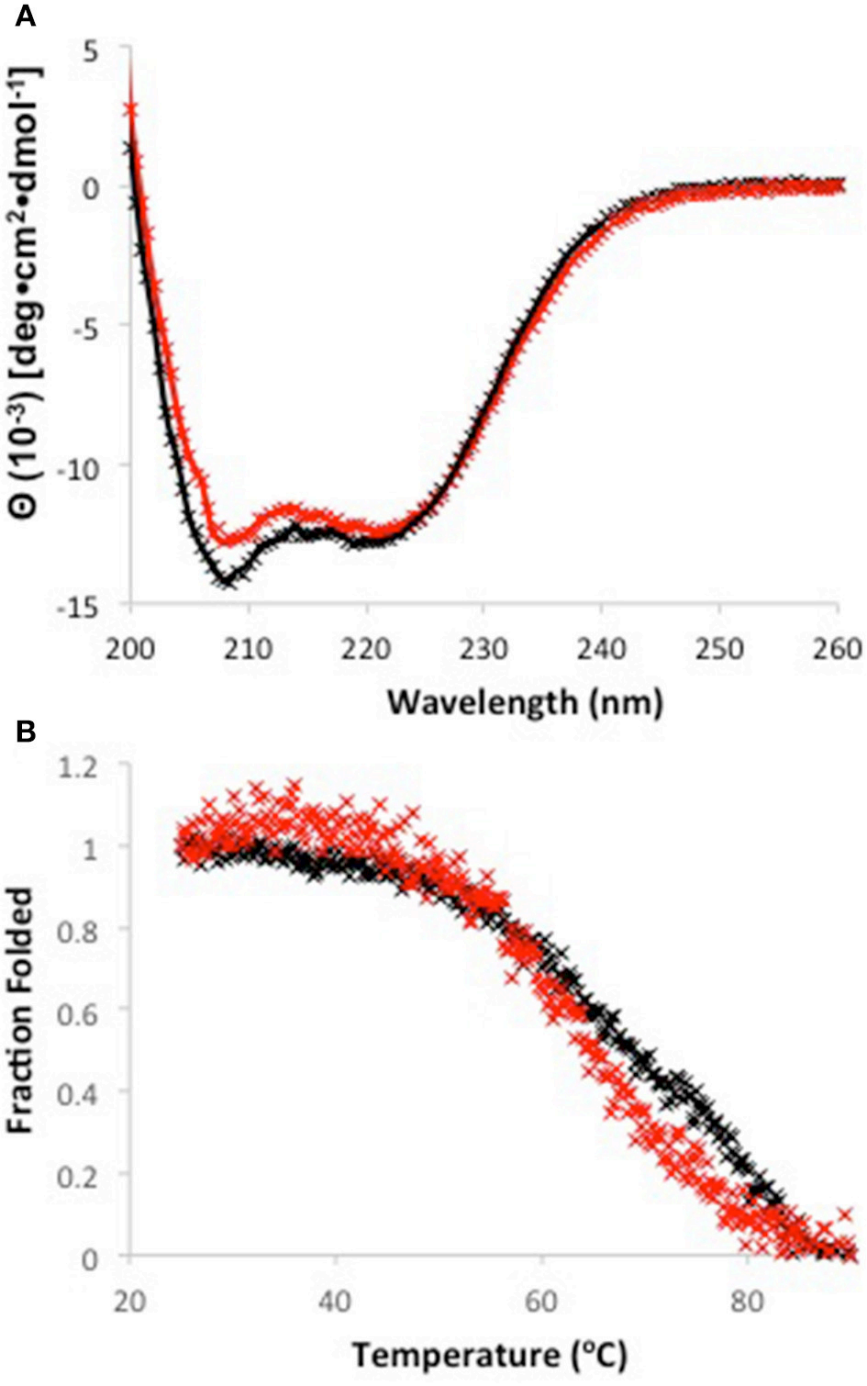
**(A)** Circular dichroism spectra for apo Zur in the absence (black) and presence (red) of 15 μM Zn. Data points are shown as (x) and fits are shown as solid lines. **(B)** Melt curves measured at 208 nm for apo (black) and holo (red) Zur.

*E. coli* Zur also binds two zinc ions, one at a high affinity site composed of 4 Cys residues (A site) and one at a low affinity site composed of 3His, 1Glu (B site; Outten et al., 2001; Gilston et al., 2014). Alignment of the two sequences (Figure 4) indicates 28% sequence identity and complete conservation of zinc ligands at both sites. Although the binding sites are conserved, the affinity for zinc seems to be much lower for *P. denitrificans* Zur than for its homologs in *E. coli* (Outten and O'Halloran, 2001), *B. subtilis* (Ma et al., 2011), and *Streptomyces coelicolor* (Shin et al., 2011), which sense free zinc concentrations in the femtomolar range. Given this unusual value, we evaluated proper protein folding and stability of both apo and holo Zur using circular dichroism (CD; Figure 5). The CD spectra for the two forms of Zur were similar and fit very well to simulations indicating ~55% alpha helix, 20% beta sheet secondary structure. This is consistent with the crystal structures for other Zur homologs (Shin et al., 2011; Gilston et al., 2014). CD was also used to determine thermal stability. The presence of zinc again had very little effect, with Tm ~65°C for both apo and holo Zur. These results show that Zur is properly folded and relatively stable, suggesting that the low zinc affinity for *P. denitrificans* Zur is an authentic property of this protein. This may be related to the observation that the relatively high concentration of zinc in Zn-depleted media is sufficient for complete derepression of zinc ABC transporter genes. Although the zinc requirements for this organism are not well-defined, it should be noted that zinc supplementation of 2.5 μM was required for optimal growth of *P. denitrificans* under anaerobic conditions (Hahnke et al., 2014). Further studies will be required to determine whether the set point for zinc is elevated in this organism relative to other bacteria.

### Identification and confirmation of the Zur consensus motif

**Figure 6:**
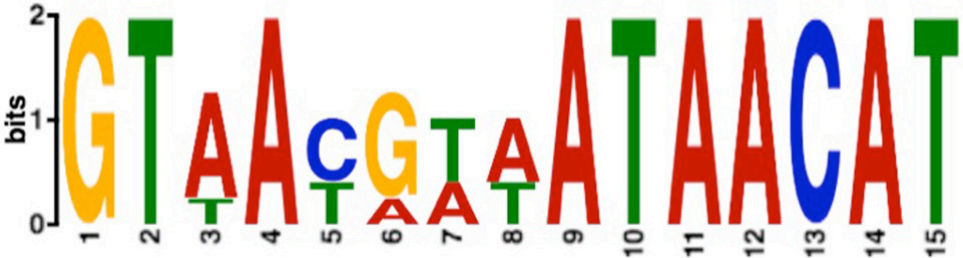
**The Zur consensus motif as determined by comparison of promoter DNA for strongly upregulated genes using MEME**. Larger letters indicate more frequent usage in the motif.

Sequences upstream of highly upregulated genes were searched for conserved sequence motifs using MEME (Bailey et al., 2006). This analysis identified a 15 bp palindromic sequence very similar to the predicted Zur motif in the *Agrobacterium* group of proteobacteria (Panina et al., 2003; Figure 6). MAST (Bailey and Gribskov, 1998) was then used to search the genome of *P. denitrificans* for this motif. Only five motifs were identified with positional *p* < 0.01, with one located in each of the promoters of the COG0523 gene (pden1338), the cluster C ABC transporter (pden1341) and the cluster A-I ABC transporter *aztA* (pden1595). The *zur* (pden4139) and *znuA* (pden4140) genes are divergently transcribed and the promoter region for these two genes contains the remaining two Zur motifs (Table 7). All of these genes are significantly upregulated under zinc limitation (Table 1). Approximately 150 base pairs (bp) of DNA was amplified from each of these promoter regions for use in EMSA experiments to verify specific Zur binding. Although MAST did not identify a Zur motif in the promoter of *norC*, a manual inspection identified a weakly conserved motif upstream of this gene (Table 7). Given the significant upregulation of *norCB* genes under zinc limitation and the unusual nature of this observation, *norC* promoter DNA was also prepared for EMSA experiments.

**Table 7:** **Locations and sequences of conserved Zur motif on various gene promoters**.

**Gene**	***p*-value**	**5′ to 3′ Sequence**	**Position**
pden1338	9.4 × 10^−5^	GTAACGTAATAACAT	19–33
pden1341	7.2 × 10^−4^	GTAATATAATAACAT	26–40
pden1595	1.4 × 10^−4^	GTAATGTAATAACAT	30–44
pden4139	3.1 × 10^−4^	GTAACGATATAACAT	2–16
pden4140	8.1 × 10^−4^	GTTACGATATAACAT	36–50
pden2484	nd	TATCGGAAATAACAG	17–31

*The p-value indicates the likelihood of a single random subsequence of the length of the motif scoring at least as well as the observed match. The position is the number of base pairs upstream of the translational start site for the conserved motif. Nd, not detected by MAST search*.

**Figure 7:**
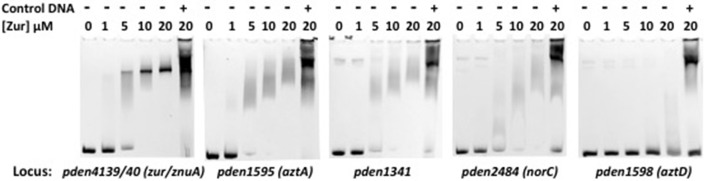
**EMSA results for Zur binding to promoter DNA**. One hundred twenty to one hundred fifty bp of promoter DNA (0.1 μM) was incubated with increasing concentrations of Zur in the presence of 25 μM Zn and 100 μM EDTA. At the highest Zur concentration, a 5-fold excess of salmon sperm DNA (50 μg/ml) was included to test the specificity of promoter binding.

For each EMSA experiment, 0.1 μM of promoter DNA was incubated with increasing concentrations of Zur protein in the presence of 25 μM ZnCl_2_ and 100 μM EDTA. Samples were run on native polyacrylamide gels and the DNA visualized by ethidium bromide staining. To evaluate specific binding, a 5-fold excess of non-specific salmon sperm DNA was included at the highest Zur concentration. There are 71 bp of non-coding DNA between *aztC* and *aztD* genes with a nearly perfect inverted repeat of sequence GGAgAcTtTCC at −11 to −1 from the *aztD* translation start site. This sequence does not match the Zur box consensus. This, combined with the significantly greater induction of *aztD* than other *azt* genes, suggests that *aztD* is regulated separately from the rest of the operon. Thus, this region of DNA was used as a negative control for specific Zur binding. The results show that Zur begins to retard each target DNA at ~5 uM whereas negative control DNA does not begin to shift until 20 μM Zur have been added (Figure 7). Moreover, non-specific salmon sperm DNA can completely displace the negative control DNA but not target DNA, allowing us to differentiate between specific and non-specific binding.

It should be noted that target DNA was progressively shifted with increasing Zur concentration in every case except for *zur/znuA* (pden4139/40). This has been observed for other Zur homologs and attributed to binding of multiple Zur dimers on the DNA probe at increasing concentration (Shin et al., 2007; Li et al., 2009). The fact that the *zur/znuA* (pden4139/40) promoter contains two Zur motifs may mask this effect, as two Zur dimers would presumably bind with high affinity, causing a large and precise shift. In any case, the gradual shifting allows for a relative evaluation of DNA binding affinity and indicates that Zur binds these promoters in order of decreasing affinity: *zur/znuA* > *aztA* ~ *pden1341* > *norC*.

**Figure 8:**
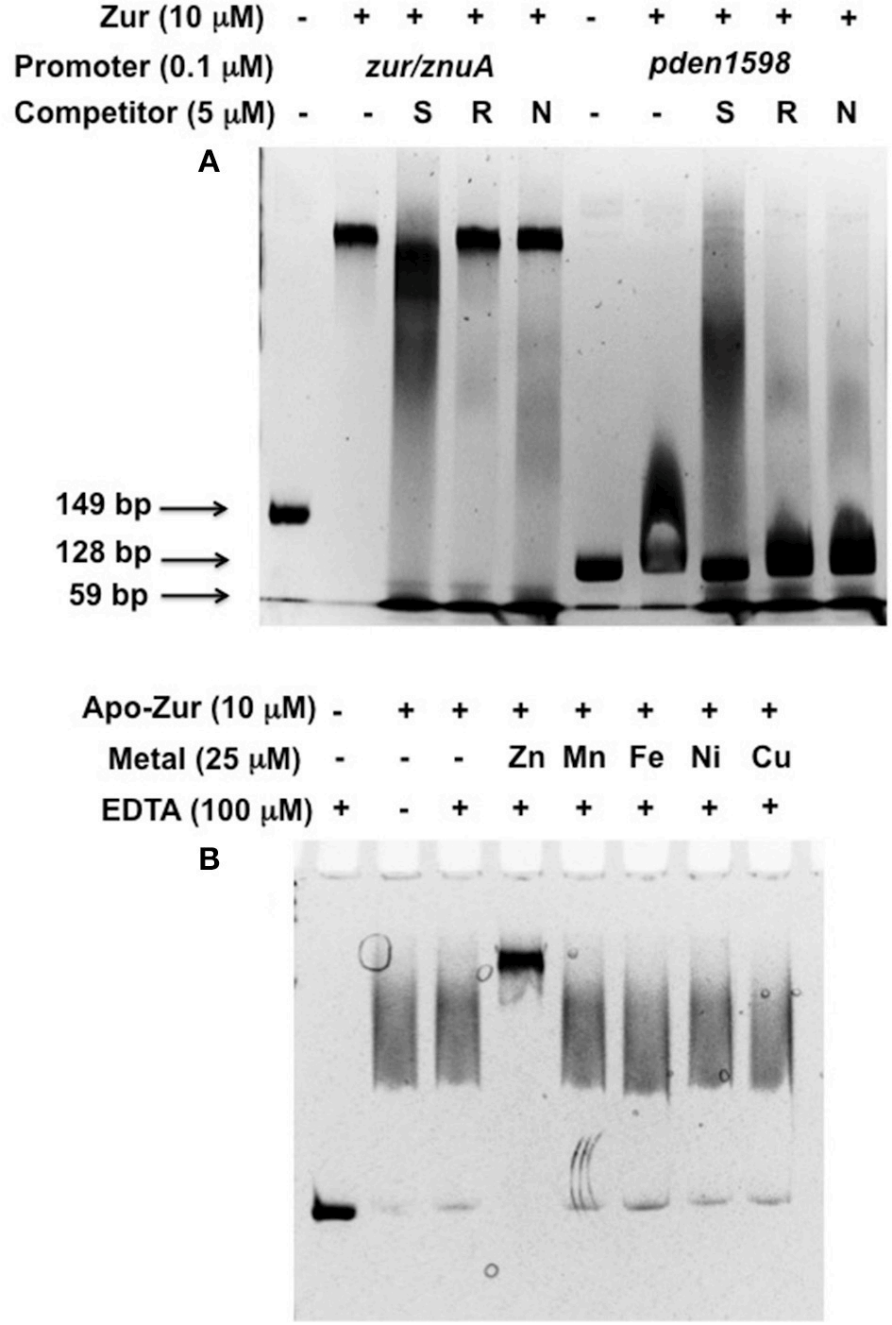
**EMSA results for specific DNA and metal binding by Zur**. **(A)** 0.1 μM of 149 bp *zur/znuA* (lanes 1–5) or 128 bp *aztD* (lanes 6–10) promoter DNA was combined with 10 μM Zur, 25 μM Zn, and 100 μM EDTA in the presence or absence of a competitor oligomer of 59 bp at 5 μM containing the specific *znuA/zur* sequence (S), a randomized sequence (R) or the *norC* sequence (N). **(B)** 10 μM apo-Zur was used as is or incubated with 25 μM of the indicated metal prior to addition of 100 μM EDTA and 0.1 μM *znuA/zur* promoter DNA.

We next set out to confirm the DNA sequence specificity of the Zur interaction as well as its metal requirements. The sequence specificity was further confirmed by competition with specific and random DNA oligomers (Figure 8A). Under the same conditions as previously, 10 uM Zur was combined with 0.1 uM *zur/znuA* (149 bp) or *aztD* (128 bp) promoter DNA in the presence and absence of specific or random 59 bp oligomer competitors at 5 uM. The specific competitor includes both Zur motifs in the *zur/znuA* promoter while the random competitor is a scrambled sequence of the same length and nucleotide composition (Figure S1). We also evaluated the effect of a 59 bp oligomer identical to the *norC* promoter region. The results show that the large shift of *zur/znuA* promoter DNA in the presence of Zur is disrupted although not completely displaced by the specific competitor. Neither the random nor the *norC* competitor appeared to have any significant effect. Similarly, the weakly bound *aztD* promoter is completely displaced by the specific competitor and appears largely disrupted by both the *norC* and random competitors. For metal specificity requirements, all EMSA buffer conditions were identical to those used previously except that zinc was omitted and apo Zur was used. Each reaction contained 0.1 uM *zur/znuA* promoter DNA, 10 uM apo Zur and Zn^2+^, Mn^2+^, Fe^2+^, Ni^2+^, or Cu^2+^, at 25 uM was added prior to EDTA at 100 uM. Equivalent weak binding is observed for the apo protein in the absence and presence of all metals except zinc. Addition of zinc results in a strong shift, indicating tight DNA binding for Zur only in the presence of the appropriate metal (Figure 8B). These experiments confirm both DNA sequence specificity and metal specificity for the Zur/DNA binding interaction.

Although the Zur motif appears in the promoters of the most highly expressed operons (Table 7), there are many genes identified by RNA-seq that lack Zur motifs. This may be due to some promiscuity of Zur binding. Alternatively, these may be regulated by other zinc-responsive transcription factors. We have identified a *zntA* homolog that is differentially expressed during zinc starvation, likely through the action of ZntR. Further, four transcriptional regulators in addition to Zur were found to be differentially expressed during zinc limitation. Therefore, it seems likely that there is significant crosstalk between zinc homeostasis and other transcriptional regulation pathways as has been suggested for *Acenitobacter baumannii* (Mortensen et al., 2014).

It is of no surprise that the Zur regulon in *P. denitrificans* includes the zinc ABC transporters *znuABC* and *aztABCD*. Zinc ABC transporters have been found to be regulated by Zur in virtually every gram-negative species studied. The direct regulation of a cluster C (or cluster 5) ABC transporter by Zur is somewhat unusual. An association between zinc and nickel homeostasis has been observed in *S. pneumoniae* (Manzoor et al., 2015). AdcR, which is structurally unrelated to Zur (Guerra et al., 2011), acts as the zinc-dependent transcriptional repressor in this organism (Shafeeq et al., 2011). Intriguingly, zinc and nickel were found to have opposite effects on AdcR-regulating genes, with nickel leading to derepression. EMSA further showed that nickel could compete with zinc and disrupt AdcR binding to promoter DNA (Manzoor et al., 2015). However, we find that nickel has no impact on Zur DNA binding (Figure 8B). Further, it should be noted that cluster C transporters may be specific for various solutes other than nickel (Berntsson et al., 2010), and a precise assignment of specificity in this transporter has not been made. An *in vitro* analysis of metal binding to the protein encoded by pden1341 would be of interest to assess the possibility that it may function in zinc import. Finally, the sheer number of metal ABC transporters upregulated during zinc deprivation is startling. The majority of these are predicted iron or cobalamin transporters, which are typically regulated by the Zur-related ferric uptake regulator (Fur). Crosstalk between the Zur and Fur regulons has also been observed previously (Mazzon et al., 2014) and may be at work here, given the relatively modest induction of these genes during zinc limitation.

Perhaps the most surprising result of this study is the upregulation of genes involved in denitrification and microaerobic metabolism during zinc deprivation. To our knowledge, the only other report of zinc deprivation influencing denitrification comes from *Pseudomonas aeruginosa* where the nitrite reductase operon *nirCFGHJL* was downregulated ≥ 2-fold in a *znuA* mutant defective in zinc acquisition (Pederick et al., 2015). This observation was only briefly mentioned and not further explored. Although the promoter of *norC* (pden2484) contains a poorly conserved Zur motif, Zur still binds to promoter DNA with greater affinity than our negative control (pden1598), albeit with weaker apparent affinity than the ABC transporter promoters. This weaker affinity was also apparent from competition experiments. In *B. subtilis*, temporal regulation of various genes has been observed as a consequence of differential binding of promoter sequences by Zur in the non-fully metallated form (Zur_2_Zn_3_; Shin and Helmann, 2016). The presence of EDTA in our EMSA assays may yield such a form of Zur that is inefficient in binding the *norC* promoter but efficient in binding the *zur/znuA* promoter, suggesting that *norC* would be derepressed earlier during Zn limitation. Further studies will be required to validate this assertion. Nevertheless, RNA-seq and EMSA experiments suggest that the *norC/B* genes are directly regulated by Zur in response to zinc limitation in *P. denitrificans*.

There is no obvious functional link between zinc homeostasis and denitrification or anaerobic metabolism. Equally puzzling is the observation of *norCB* and *nirS* expression under aerobic conditions in media where ammonia was the sole nitrogen source. However, *P. denitrificans* is capable of nitrification (Crossman et al., 1998) as well as denitrification, and both pathways may be active simultaneously under aerobic conditions (Robertson et al., 1988). Therefore, nitrite formed as a product of ammonia oxidation (nitrification) could be reduced by denitrification. The simultaneous activity of both pathways has been proposed as a means to overcome a “bottleneck” in the cytochrome chain passing electrons to oxygen (Robertson et al., 1988; Stouthamer et al., 1997). Perhaps zinc deprivation limits the ability of the organism to deal with oxidative stress generated as a result of such a bottleneck. Antioxidant functions for zinc are well-established in eukaryotes (Oteiza, 2012) and have been observed in prokaryotes as well (Gaballa and Helmann, 2002; Smith et al., 2009). This might explain the need to upregulate alternate electron transfer pathways in response to zinc limitation. Alternatively, upregulation of denitrification genes may be an indirect effect of zinc limitation. Expression of *norC, nirS, ccoG*, and *ccoN* identified in this study are also positively regulated under anaerobic conditions by proteins of the FNR (fumarate and nitrate reductase) family (Stouthamer et al., 1997), and an FNR box is present in the promoter of each (Hoeren et al., 1993; de Boer et al., 1994; Van Spanning et al., 1995; Stouthamer et al., 1997). FNR proteins may respond to cellular redox status (Van Spanning et al., 1997), which may be altered by zinc limitation as described above. Clearly, further experiments, such as measurements of denitrification activity in Zn-depleted vs. Zn-replete media, will be required to elucidate the physiological function of regulatory crosstalk between zinc limitation and denitrification.

## Authors contributions

DN and BJ performed all experiments excepting RNA sequencing. AS, TR, and FS performed RNA sequencing and preliminary analysis. EY conceived the study and wrote the paper.

## Funding

This work was supported by New Mexico IDeA Networks of Biomedical Research Excellence (NM-INBRE) funding through the National Institute of General Medical Sciences grant 8P20GM103451-12. Additional funding was provided under award number SC2 GM111170-01 from the National Institutes of Health (NIH).

## Conflict of interest statement

The authors declare that the research was conducted in the absence of any commercial or financial relationships that could be construed as a potential conflict of interest.
