# Supplementary material for: Zinc-Dependent Transcriptional Regulation in Paracoccus denitrificans
Source: Front Microbiol. 2017 Apr 11;8:569. doi: 10.3389/fmicb.2017.00569 (PMC5387054; doi:10.3389/fmicb.2017.00569)
Supplement: Supplementary file 3 [file DataSheet3.DOCX]

Supplemental Information for “Zinc-dependent Transcriptional Regulation in Paracoccus denitrificans”

Durga P. Neupane, Belkis Jacquez, Anitha Sundararajan, Thiruvarangan Ramaraj, Faye D. Schilkey and Erik T. Yukl*

**Table. S1:** DNA primers used for qPCR and EMSA experiments

| **Locus** | **Gene** | **RT-qPCR primer** | **EMSA primer** |
| --- | --- | --- | --- |
| **pden0970** | ***dnaN*** | **Fwd: 5’-ATGTCGTGGGTCAGCATA-3’** |  |
|  |  | **Rev: 5’-GATGCCGTTCAGATAATAGC-3’** |  |
| **pden2484** | ***norC*** | **Fwd: 5’-CTGAAGGGCTGGATGGAAT-3’**  **Rev: 5’-TTCTGGGTCTTGATCGTTCC-3’** | **Fwd: 5’- TTCCGGACAAAATGTCCAGCT-3’**  **Rev: 5’- TAGAAGACGTTCCGGGCCAT-3’** |
| **pden2487** |  | **Fwd: 5’-ACCTTTGGATGAAGGAACCC -3’**  **Rev: 5’-GTCCATGATGACGTATTGCG-3’** |  |
| **pden4169** |  | **Fwd: 5’-ATCGACCTGTCGGTGGTG-3’**  **Rev: 5’-TCCGTCATCGTATTGACCAG-3’** |  |
| **pden0319** |  | **Fwd: 5’-GGTGATCTTCATCGCCATCT-3’**  **Rev: 5’-GCGAAGCCGACATAGGTAAA -3’** |  |
| **pden1595** | ***aztA*** |  | **Fwd: 5’- AATCACAAAACCGGCGCCTC-3’**  **Rev: 5’- GGTTCTGGGCATCACTGGAT-3’** |
| **pden1597** | ***aztC*** |  | **Fwd: 5’- GTCTATCTCGTCTCGCTTCTG-3’**  **Rev: 5’- CGTCATGATCGAACATGTG-3’** |
| **pden1598** | ***aztD*** |  | **Fwd: 5’- GCCGCCCGCTGACCAGATTT-3’**  **Rev: 5’- AAGGTCAGGGCAAGGGCGCT-3’** |
| **pden1341** |  |  | **Fwd: 5’- AAGAGAGGGTTGAGCCTGCC-3’**  **Rev: 5’- AAGCGAGCAGTTTGCAGAGC-3’** |
| **pden4140** | ***znuA*** |  | **Fwd: 5’- CGTGAAACGGGTCAGGCGG-3’**  **Rev: 5’-CGGCAAGGACAAGGGAAGAAGG-3’** |

1. **Specific EMSA Competitor**

5’-GGGCATGATGTTATATCGTTACATTTTTGCTTGACGGGTTACGATATAACATAAATAAC-3’

3’-CCCGTACTACAATATAGCAATGTAAAAACGAACTGCCCAATGCTATATTGTATTTATTG-5’

1. **Random EMSA Competitor**

5’- ATTAACAAGTGTGTACTTGTTTTATCATCTCACAGTTAAAGTCGGGAAAATAGGAGCCA-3’

3’- TAATTGTTCACACATGAACAAAATAGTAGAGTGTCAATTTCAGCCCTTTTATCCTCGGT-5’

1. ***norC* EMSA Competitor**

5’-GACTCACGCTTGCCGGACAGTGAGGTCTTATCGGAAATAACAGATAGGGGACCCGCGTC-3’

3’-CTGAGTGCGAACGGCCTGTCACTCCAGAATAGCCTTTATTGTCTATCCCCTGGGCGCAG-5’

**Figure S1:** Sequences for specific and random oligomers used as EMSA competitors (Fig. 8A). The predicted Zur motifs are indicated in red text.
